# Lung ultrasound–guided positioning strategy for the prevention of ventilator-associated pneumonia in neonates

**DOI:** 10.3389/fped.2026.1765924

**Published:** 2026-02-02

**Authors:** Zhenyu Liang, Binghui Li, Xiao Zhang, Lin Li, Debo Xu, Qiong Meng, Xihua Huang, Huiyi Li

**Affiliations:** Department of Pediatrics, The Affiliated Guangdong Second Provincial General Hospital of Jinan University, Guangzhou, China

**Keywords:** infection prevention, lung ultrasound, neonate, positioning, randomized controlled trial, ventilator-associated pneumonia

## Abstract

**Objective:**

To determine whether a lung ultrasound–guided positioning strategy reduces the incidence of ventilator-associated pneumonia (VAP) in mechanically ventilated neonates compared with conventional empiric repositioning.

**Data sources:**

A prospective, randomized controlled trial.

**Interventions:**

In this prospective, randomized controlled trial, term neonates requiring invasive mechanical ventilation for more than 48 h were enrolled in the Level III NICU of Guangdong Second Provincial General Hospital from December 2023 to December 2025. Eligible infants were randomly assigned (1:1) to receive either conventional positioning or LUS-guided positioning management. The LUS-guided group underwent twice-daily bedside ultrasound assessments to detect atelectasis or consolidation, and posture was adjusted accordingly to optimize dependent drainage and ventilation–perfusion matching. The primary outcome was the incidence of VAP. Secondary outcomes included duration of mechanical ventilation, feeding intolerance, pulmonary hemorrhage, pneumothorax, new-onset intracranial hemorrhage, mortality, and NICU length of stay.

**Results:**

A total of 94 neonates were randomized (47 per group), and 89 completed follow-up. Baseline characteristics were comparable between groups. The incidence of VAP was significantly lower in the LUS-guided group than in the control group (17.8% vs. 38.6%, *p* = 0.017), with an unadjusted odds ratio (OR) of 0.28 (95% CI, 0.13–0.90) and an adjusted OR of 0.32 (95% CI, 0.12–0.86, *p* = 0.024). The LUS-guided group also showed a shorter duration of mechanical ventilation [median (IQR): 11.0 (9.0–13.0) vs. 12.0 (10.0–14.0) days, *p* = 0.023], with a mean difference of −1.4 days (95% CI, −2.6 to −0.2). No significant differences were observed in other secondary outcomes, including pulmonary hemorrhage, pneumothorax, feeding intolerance, new intracranial hemorrhage, or mortality (all *p* > 0.05).

**Conclusion:**

Lung ultrasound–guided positioning significantly reduced the incidence of ventilator-associated pneumonia and shortened ventilation duration without increasing adverse events. This physiologically informed, radiation-free approach enables individualized postural management to optimize lung aeration and secretion clearance. LUS-guided care is feasible, safe, and easily integrated into NICU workflows, providing a promising adjunct to standard VAP prevention bundles. Further multicenter, adequately powered trials and cost-effectiveness studies are warranted to confirm these findings and support broader clinical adoption.

## Introduction

Ventilator-associated pneumonia (VAP) is one of the most frequent and serious hospital-acquired infections in neonatal intensive care units (NICUs), leading to prolonged mechanical ventilation, extended hospitalization, and increased mortality. The incidence of neonatal VAP varies substantially across surveillance definitions and patient populations, ranging from 1.4 to 7 episodes per 1,000 ventilator-days in high-income countries and up to 89 episodes per 1,000 ventilator-days in developing regions (1, 2).

Preterm and very-low-birth-weight infants are especially vulnerable due to immature immune defense, impaired mucociliary clearance, and prolonged exposure to invasive ventilation (3).

The pathogenesis of neonatal VAP involves microaspiration of contaminated secretions, colonization of endotracheal tube biofilms, and gravity-dependent pooling of airway secretions (4). Improper or static body positioning may exacerbate dorsal lung collapse and secretion retention, leading to ventilation–perfusion mismatch, hypoxemia, and bacterial proliferation. Studies in adults and pediatric patients have shown that appropriate positioning interventions—such as semi-recumbent or prone postures—can improve oxygenation and may reduce VAP incidence (5, 6). However, these strategies have not been adequately standardized or validated in neonates, whose thermoregulatory immaturity and hemodynamic instability restrict frequent posture adjustments (7, 8).

Lung ultrasound (LUS) has emerged as a reliable, radiation-free bedside imaging tool for dynamic assessment of pulmonary aeration, atelectasis, and consolidation in neonates (9, 10). Quantitative LUS scoring enables real-time assessment of regional ventilation and early detection of asymmetric aeration (11, 12). Integrating LUS findings into positioning management provides a physiologically guided strategy to optimize lung recruitment and secretion clearance before irreversible infection develops.

However, evidence supporting this approach in the neonatal population remains limited. Therefore, this study aimed to evaluate the clinical efficacy of a lung ultrasound–guided positioning intervention strategy in preventing ventilator-associated pneumonia among neonates receiving invasive mechanical ventilation. We hypothesized that LUS-guided individualized positioning would reduce the incidence of neonatal VAP and improve respiratory outcomes compared with routine nursing positioning.

## Materials and methods

### Trial design, setting, and ethical approval

This prospective randomized controlled trial was conducted in the Level III Neonatal Intensive Care Unit (NICU) of the Affiliated Guangdong Second Provincial General Hospital of Jinan University. The study protocol was reviewed and approved by the Institutional Ethics Committee of Guangdong Second Provincial General Hospital (Approval No. 2023-KY-KZ-266-02).The study was registered with the Chinese Clinical Research Registry (MR-44-25-000829) and on ClinicalTrials.gov (NCT07254507; registered November 25, 2025).Written informed consent was obtained from the parents or legal guardians of all participating neonates. The study was conducted in accordance with the ethical principles of the Declaration of Helsinki.

### Patient characteristics

Eligible participants included term neonates (gestational age 37–42 weeks) who required invasive mechanical ventilation for more than 48 h. Upon fulfilling this criterion (i.e., after completing >48 h of ventilation) and meeting all other inclusion/exclusion criteria, eligible infants were formally enrolled and randomized. Patients were enrolled between December 2023 and December 2025. Exclusion criteria included preterm neonates (This decision was made primarily to control for significant confounding factors inherent to prematurity, such as respiratory distress syndrome, bronchopulmonary dysplasia, hemodynamic instability related to patent ductus arteriosus, and a higher baseline risk of intracranial hemorrhage. These conditions profoundly influence both the risk of VAP and key respiratory outcomes, and their inclusion would have introduced substantial heterogeneity, complicating the interpretation of the intervention's specific effect. Furthermore, the safety and feasibility of applying a standardized, frequent positioning protocol in physiologically fragile preterm infants required separate evaluation. This study was therefore designed as a proof-of-concept investigation in a more homogeneous, term population to first establish efficacy and safety before future adaptation for preterm infants); neonates with early-onset sepsis or pre-existing pneumonia; patients unfit for enteral intake; neonates requiring mechanical ventilation for surgical causes; and infants with multiple congenital anomalies or suspected chromosomal disorders.

### Randomization and blinding

Random allocation (1:1) to the conventional or LUS-guided positioning arm was performed by an independent biostatistician through a computer-generated randomization list. The biostatistician was not involved in the design, clinical execution, or outcome analysis of the trial. Allocation concealment was guaranteed by using sequentially numbered, opaque, sealed envelopes, opened only following written informed consent. The radiologists who interpreted all chest radiographs and the senior neonatologists who adjudicated the final diagnosis of VAP (based on the CDC criteria integrating clinical, laboratory, and radiographic findings) were blinded to the patient's study group allocation. This was to minimize detection bias in the assessment of the primary outcome.

### Intervention

#### Baseline assessment prior to randomization

Upon initiation of invasive mechanical ventilation and before any study-related procedures, all neonates underwent a standard diagnostic evaluation that included an anteroposterior chest radiograph. This radiograph was used to diagnose the underlying respiratory condition (e.g., meconium aspiration syndrome, transient tachypnea of the newborn, pneumonia) and confirm the need for ongoing ventilatory support. Lung ultrasound was not employed as a diagnostic tool at this pre-randomization stage, ensuring a uniform baseline assessment across both study groups.

All infants were invasively ventilated via endotracheal tube using a SLE6000 mechanical ventilator equipped with a heated humidification system and disposable ventilator circuits. Both groups received routine NICU care, including standardized VAP preventive measures, antibiotic therapy when indicated, and general nursing management according to hospital policy and international guidelines. Antibiotic selection and adjustment followed local antimicrobial stewardship recommendations based on culture and sensitivity results.

#### Control group (conventional positioning management)

Infants in the control group were repositioned every two hours, alternating among the supine, left lateral, right lateral, and prone positions (13). The head of the bed was elevated by 15°–30°, with the body kept in a slightly flexed posture—hips aligned along the midline, shoulders slightly protracted, and head maintained at midline with unrestricted arm movement. Repositioning was performed only when vital signs were stable and deferred during resuscitation, ventilator adjustments, intravenous infusions, or deep sleep. If heart rate fluctuated by >20 bpm or SpO₂transiently dropped below 90% (excluding airway obstruction), the interval between position changes was extended to 3–4 h. No ultrasound evaluation was performed in this group.

#### LUS-guided group (lung ultrasound–guided positioning)

Positioning was guided by the principles of dependent drainage of affected lung regions, optimization of aeration in healthier areas, and prevention of pressure-related injury (14, 15). In addition to standard care, LUS assessments were conducted twice daily at fixed time points (08:00 and 18:00) to evaluate regional aeration and guide individualized postural adjustments:

- When unilateral atelectasis or pulmonary edema was identified, the dependent lateral position was maintained for approximately 1 h, followed by contralateral or prone positioning for 3 h.
- For predominantly anterior lesions, prone positioning was shortened to 1 h, and supine or lateral positions extended to 3 h.
- For posterior lesions, supine or lateral positions were shortened to 1 h, and prone positioning extended to 3 h.
- Once consolidation or edema resolved, conventional two-hour rotation was resumed.

If SpO₂persistently remained below 90% (excluding handling or feeding interference), respiratory rate increased by >20 breaths/min from baseline, or airway secretions markedly increased, additional LUS assessments were performed.

All ultrasound examinations were performed by two trained pediatricians using a Philips CX50 color Doppler ultrasound system with a high-frequency linear probe (frequency range: 8–12 MHz), and images were reviewed by a senior neonatologist experienced in lung ultrasound for quality assurance. (Representative lung ultrasound images illustrating the detection of pneumonia and the subsequent changes following guided positional therapy are provided in Supplementary Figures S1, S2). All physicians underwent biannual hands-on critical care ultrasound training conducted by certified international instructors to ensure diagnostic consistency and technical competence.

### Study definitions

All enrolled neonates were prospectively observed for the development of VAP. The diagnosis of VAP followed the criteria established by the Centers for Disease Control and Prevention (CDC) (15), applicable to infants under one year of age who had received invasive mechanical ventilation for more than 48 h. A diagnosis required evidence of new or progressive pulmonary infiltrates on chest imaging accompanied by worsening gas exchange.

In addition, at least three of the following clinical or laboratory findings were necessary: temperature instability (rectal temperature >38℃ or <35.5℃), leukopenia (WBC <4 × 10⁹/L) or leukocytosis (WBC >15 × 10⁹/L) with a left shift (>10% band forms), appearance or increase of purulent tracheal secretions, change in sputum characteristics, increased secretion volume requiring more frequent suctioning, apnea, signs of respiratory distress (tachypnea, retractions, grunting, nasal flaring), adventitious breath sounds (wheezing, rales, or rhonchi), bradycardia (<100 bpm), or tachycardia (>170 bpm).

### Diagnosis and adjudication of VAP

The diagnosis of VAP was established prospectively for all enrolled neonates based on the standardized criteria from the Centers for Disease Control and Prevention (CDC) for infants <1 year of age (15). The diagnosis required all of the following components:

- Radiographic Evidence: New or progressive pulmonary infiltrates on chest radiograph.
- Clinical & Laboratory Evidence: At least three of the following: temperature instability, leukopenia or leukocytosis with left shift, new onset of purulent tracheal secretions or increased suctioning requirements, apnea or worsened respiratory signs (e.g., tachypnea, retractions), adventitious breath sounds, or bradycardia/tachycardia.

Adjudication Process with Blinding: To minimize detection bias, the adjudication process was structured as follows:

- Chest Radiograph Interpretation: All chest radiographs were interpreted by staff radiologists who were blinded to the patient's study group allocation and clinical details beyond the requisition indication.
- Final VAP Adjudication: A panel of two senior neonatologists, also blinded to treatment allocation, independently reviewed all relevant data for each suspected case. This included the blinded radiologist's report, daily clinical notes, laboratory results (complete blood count, C-reactive protein), and microbiology reports from tracheal aspirate or bronchoalveolar lavage cultures. A diagnosis of VAP was confirmed only upon consensus between the two adjudicators, strictly according to the CDC criteria outlined above. In case of disagreement, a third blinded senior neonatologist was consulted for final decision.

### Data gathering

After admission and enrollment, detailed antenatal, perinatal, and postnatal histories were obtained, followed by a comprehensive physical examination. Gestational age and admission weight were recorded for each neonate. Laboratory investigations included a complete blood count (CBC), C-reactive protein (CRP), liver and renal function tests, capillary blood gas (CBG) analysis, automated blood culture, and non-bronchoscopic bronchoalveolar lavage (NB-BAL) for culture and sensitivity. The control group and the LUS-guided group followed different imaging protocols for pulmonary monitoring. The control group received scheduled chest radiographs (anteroposterior and lateral views) on the 1st, 3rd, and 7th days of mechanical ventilation, repeated as clinically indicated. Conversely, the LUS-guided group did not undergo scheduled radiographs; instead, pulmonary monitoring was based on twice-daily bedside lung ultrasound (LUS) assessments (at 08:00 and 18:00) to guide positioning. In the LUS-guided group, chest radiographs were reserved for specific clinical indications, including acute deterioration, suspicion of complications like pneumothorax, or to aid in the formal adjudication of a suspected VAP.

### Primary and secondary outcomes

The primary outcome was the effect of lung ultrasound–guided positioning management on the incidence of ventilator-associated pneumonia in mechanically ventilated neonates.

Secondary outcomes included the occurrence of neonatal feeding intolerance, pulmonary hemorrhage, pneumothorax, new-onset intracranial hemorrhage, death, duration of invasive mechanical ventilation (calculated from initiation of ventilation until successful extubation or death), and NICU length of stay.

The sample size was calculated based on previously reported Neo-VAP incidence rates (20–35%) (16). An expected incidence of 31% in the conventional group and an absolute reduction of 25% in the LUS-guided group were considered clinically significant (17). Using a two-sided α = 0.05 and power = 80%, the minimum required sample size was 37 neonates per group. Accounting for a potential 20% attrition rate, 47 infants per group were targeted. An interim analysis was planned after 50% of participants had been enrolled, applying a two-sided symmetric O'Brien–Fleming design (18).

### Statistical analysis

Statistical analyses were performed using IBM SPSS Statistics software (version 20.0; IBM Corp., Armonk, NY, USA). Continuous variables were summarized as mean ± standard deviation (SD) or median (interquartile range, IQR) depending on data distribution, while categorical data were presented as numbers and percentages. Between-group comparisons of categorical variables were made using the Chi-square or Fisher's exact test, and continuous variables were assessed with the independent-samples t test or Mann–Whitney *U*-test as appropriate. A two-tailed *P* value <0.05 was considered statistically significant. The analysis adhered to the original study protocol, and all procedures were completed as planned.

### Study outcome analysis

**Figure 1:**
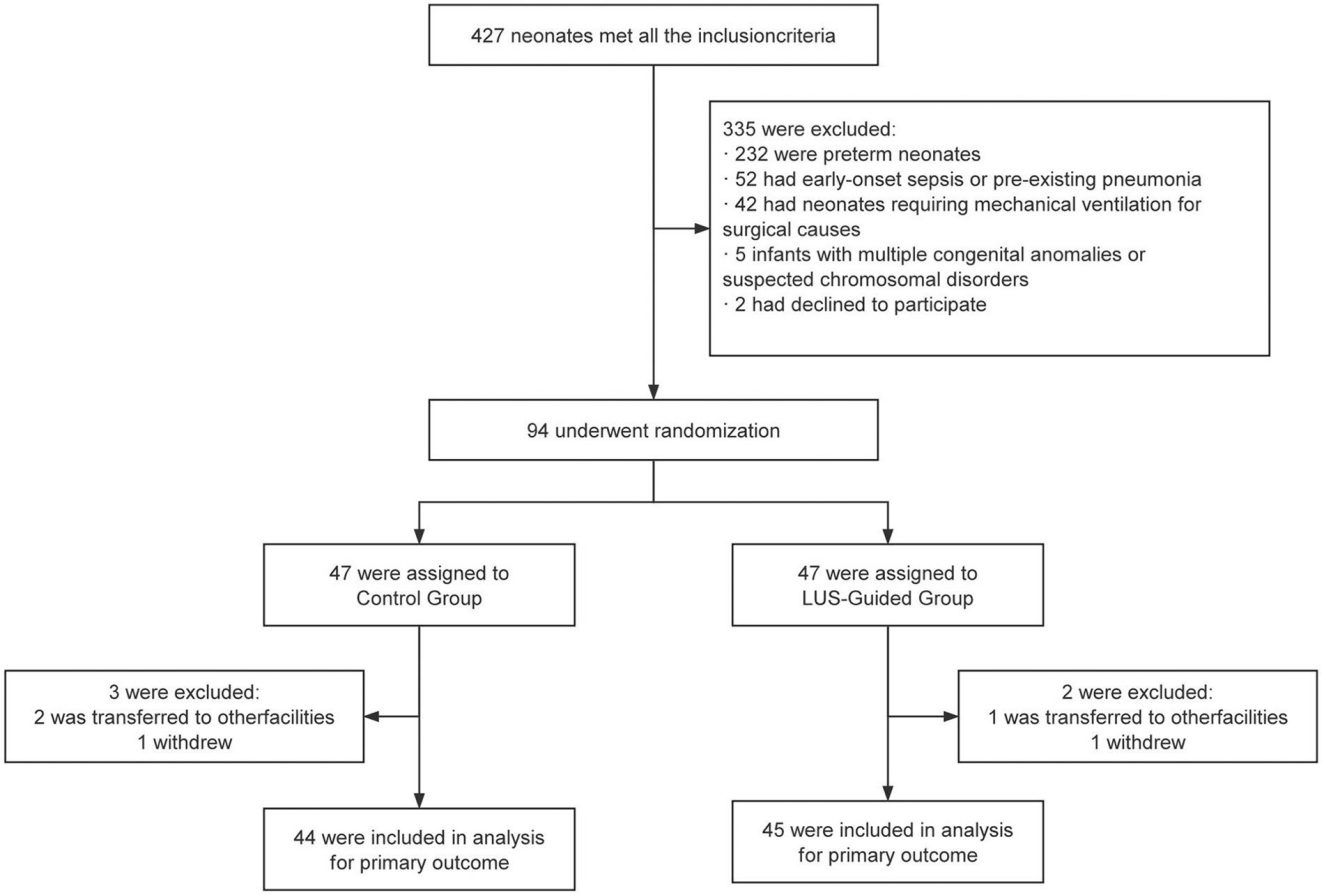
Study flow diagram. Patient enrollment and randomization.

During the study period, a total of 427 neonates required mechanical ventilation. After applying the predefined exclusion criteria, 94 patients were enrolled and randomized into two groups: LUS-guided group (*n* = 47) and control group (*n* = 47). Ultimately, 89 neonates completed follow-up and were included in the final analysis (Figure 1). The baseline demographic and clinical characteristics were comparable between the two groups, with no statistically significant differences observed in any parameter (all *p* > 0.05) (Table 1). Moreover, there was no significant difference in the distribution of various pathogen types between the two groups (all *p* > 0.05) (see Online Supplementary).

**Table 1:** Baseline criteria of the control group and LUS-guided group.

Characteristics	Control group (*n* = 44)	*n* (%)	LUS-guided group (*n* = 45)	*n* (%)	*P_value_*
Gender					
Male	30	68.2%	27	60.0%	0.421
Female	12	31.8%	18	40.0%	
Gestational age (weeks)					
Mean± SD	39.23 ± 1.72		39.51 ± 1.71		0.394
Birth weight (KG)					
Mean± SD	3.166 ± 0.33		3.10 ± 0.35		0.418
Antenatal Steroids	19	43.2%	14	31.1%	0.337
Type of delivery					
C-section	26	59.1%	28	62.2%	0.762
5 min Apgar Score					
Median (IQR)	8.00 [7.00, 9.00]		8.00 [7.00, 9.00]		0.392
SNAPPE II Score					
Mean ± SD	4.86 ± 1.05		4.91 ± 1.04		0.866
Indications of MV					
PPHN	12	27.3%	18	40.0%	0.204
TTN	6	13.6%	7	15.6%	0.798
ICH	5	11.4%	1	2.2%	0.110
HIE	18	40.9%	21	46.7%	0.584
Age at VAP (days)					
Mean ± SD	7.00 ± 1.76		7.75 ± 1.91		0.250

SD, Standard deviation; P, *p* value for comparing between the two studied groups; SNAPPE II, score for neonatal acute physiology and SNAP perinatal extension; ICH, intracranial hemorrhage; HIE, hypoxic ischemic encephalopathy; TTN, transient tachypnea of newborns; PPHN, persistent pulmonary hypertension; MV, mechanical ventilation; VAP, ventilator-acquired pneumonia.

To address potential confounding factors, a multivariate logistic regression analysis was performed to evaluate the association between LUS-guided positioning management and the incidence of VAP. After adjusting for sex, gestational age, birth weight, antenatal corticosteroid exposure, SNAPPE-II score, persistent pulmonary hypertension of the newborn, transient tachypnea of the newborn, intraventricular hemorrhage, and hypoxic-ischemic encephalopathy, LUS-guided positioning remained significantly and independently associated with a lower risk of VAP (OR = 0.32, 95% CI: 0.12–0.86, *p* = 0.024), indicating that LUS-based postural intervention was an independent protective factor against VAP. None of the other covariates reached statistical significance (Table 2).

**Table 2:** Multivariate logistic regression LUS-guided group with ventilator-acquired pneumonia.

Variable	β	OR	95% CI (lower)	95% CI (upper)	*P* value*
LUS-Guided	−1.14	0.32	0.12	0.86	0.024*
Sex	−0.03	0.97	0.35	2.65	0.949
Gestational age (weeks)	0.08	1.09	0.81	1.45	0.562
Birth weight (g)	0.0002	1.00	0.10	1.00	0.808
Antenatal steroid	0.51	1.67	0.63	4.36	0.294
SNAPPE-II score	−0.20	0.81	0.50	1.32	0.404
PPHN	−0.70	0.50	0.18	1.38	0.181
TTN	−0.70	0.50	0.13	1.87	0.304
ICH	−0.16	0.86	0.13	5.71	0.872
HIE	−0.26	0.77	0.29	2.03	0.604

* Statistically significant at *p* ≤ 0.05.

For Primary Outcome, The incidence of VAP was significantly lower in the LUS-guided group than in the control group (17.8% vs. 38.6%, *p* = 0.017), corresponding to an odds ratio (OR) of 0.28 (95% CI: 0.13–0.90) (Table 3). For secondary outcomes, the duration of mechanical ventilation was significantly shorter in the LUS-guided group compared with the control group [median (IQR): 11.00 (9.00–13.00) days vs. 12.00 (10.00–14.00) days, *p* = 0.023], with a mean difference of −1.40 days (95% CI: −2.60 to −0.20) (Table 3). No significant between-group differences were observed in neonatal feeding intolerance, pulmonary hemorrhage, pneumothorax, new-onset intracranial hemorrhage, mortality, or NICU length of stay (all *p* > 0.05) (Table 3).

**Table 3:** Primary and secondary outcomes among the two studied groups.

Outcomes	Control group (*n* = 44)	*n* (%)	LUS-guided group (*n* = 45)	*n* (%)	*P* value*	OR\MD (95% CI)
Primary outcome						
VAP incidence	17	38.6%	8	17.8%	0.028*	0.35 (0.13–0.90)
Secondary outcomes						
Duration of MV						
Median (IQR)	12 (10–14)		11 (9.00–13.00)		0.023*	−1.40 (−2.60, −0.20)
NICU stay (days)						
Median (IQR)	18 (15–20)		17 (14.00–19.00)		0.142	−1.10 (−2.70, 0.40)
Feeding intolerance	17	(38.6%)	23	51.1%	0.237	1.66 (0.71–3.86)
Pulmonary hemorrhage	6	13.6%	4	8.9%	0.512	0.62 (0.16–2.36)
Pneumothorax	7	15.9%	9	20%	0.6153	1.32 (0.44–3.93)
New-onset intracranial hemorrhage	4	9.1%	1	2.2%	0.2028	0.23 (0.02–2.12)
Death	1	2.3%	1	2.3%	1.0000	0.98 (0.06–16.13)

IQR, interquartile range; OR, odds ratio; MD, mean difference; C.I.: confidence interval.

* Statistically significant at *p* ≤ 0.05.

## Discussion

This randomized controlled trial provides novel evidence that a lung ultrasound (LUS)–guided positioning intervention significantly reduces the incidence of VAP in mechanically ventilated neonates compared with conventional empiric repositioning. The LUS-guided group exhibited a markedly lower VAP incidence (17.8% vs. 38.6%) and a shorter duration of mechanical ventilation, suggesting that real-time ultrasound monitoring of lung aeration can translate into meaningful clinical benefits. These findings underscore the potential of integrating bedside LUS into daily NICU practice as a physiologically informed, individualized approach to optimize ventilation and minimize infection risk (19, 20).

The prevention of VAP relies on minimizing microaspiration, improving secretion clearance, and maintaining alveolar aeration (16, 21). In neonates, particularly those who are preterm or critically ill, these protective mechanisms are inherently compromised due to weak cough reflexes, immature surfactant systems, and prolonged endotracheal intubation (4). Improper or static body positioning aggravates the situation by promoting dependent lung collapse, impairing mucociliary transport, and fostering a microenvironment conducive to bacterial proliferation (22). By enabling dynamic visualization of aeration patterns, LUS allows clinicians to detect evolving atelectasis or consolidation at a subclinical stage and to tailor postural adjustments accordingly (23). This early, physiology-based correction likely explains the 68% relative risk reduction in VAP observed in the current study.

Previous studies in adults and older pediatric populations have demonstrated that prone and semi-recumbent positioning can improve oxygenation and reduce VAP risk by mitigating gastroesophageal reflux and enhancing ventilation–perfusion matching (24, 25). However, in neonates, the evidence base remains limited and fragmented. Fixed-interval rotation protocols (e.g., every 2–3 h) are widely used in NICUs, yet they are empirical and fail to account for heterogeneity in pulmonary pathology across time and regions. Our findings extend this knowledge by showing that individualized positioning guided by LUS may achieve targeted “dependent drainage” of secretions and “selective recruitment” of well-aerated regions, offering a dynamic, patient-specific alternative to conventional rotation. This aligns with the broader paradigm shift toward personalized ventilation strategies in neonatal critical care (26).

The clinical utility of LUS has evolved rapidly over the past decade, expanding beyond diagnosis of respiratory distress syndrome, pneumothorax, and pleural effusion to active guidance of ventilatory and hemodynamic management (27, 28). The present study represents a translational step from diagnostic imaging to real-time interventional application. Quantitative LUS scoring enables tracking of regional aeration loss, while qualitative findings—such as the presence of B-lines, consolidations, or pleural abnormalities—inform decisions on whether to reposition, recruit, or maintain current ventilator settings (29). Importantly, this noninvasive approach circumvents radiation exposure from serial chest x-rays, aligning with global initiatives for radiation minimization in NICUs (30).

Moreover, LUS-guided care is both feasible and resource-efficient. Once competency is achieved, bedside scans require less than five minutes, and the intervention can be seamlessly integrated into existing nursing workflows. The technique's real-time feedback loop also enhances multidisciplinary decision-making, facilitating communication among neonatologists, respiratory therapists, and nursing staff. In this context, LUS serves not only as an imaging modality but as a “functional monitoring tool” bridging physiology and bedside care (31, 32).

To our knowledge, this is the first prospective randomized trial to evaluate the preventive effect of lung ultrasound (LUS)–guided positioning on ventilator-associated pneumonia (VAP) in neonates. Previous investigations have mainly utilized LUS as a diagnostic tool or for guiding surfactant administration and ventilatory adjustments (12, 19). Moreover, several studies have demonstrated that ultrasound-assessed lung aeration correlates closely with respiratory system compliance and oxygenation indices, supporting its value as a dynamic bedside monitoring modality (33). In addition, LUS has been shown to detect rapid aeration changes during recruitment maneuvers and prone positioning in adult patients (13). Our findings align with these observations and extend their implications to the neonatal population, in whom invasive monitoring options are limited and clinical deterioration can progress rapidly.

Furthermore, our study corroborates prior observational data linking improved oxygenation and fewer pulmonary complications to early detection of atelectasis using bedside ultrasound (33). The LUS-guided group in our trial required shorter ventilation support, reflecting more efficient alveolar recruitment and better secretion management. Although the reduction in NICU stay did not reach statistical significance, the direction of effect suggests potential downstream benefits on resource utilization and antibiotic exposure—two key drivers of neonatal morbidity and healthcare cost (26).

An important finding is that LUS-guided postural intervention did not increase the risk of adverse outcomes such as pulmonary hemorrhage, pneumothorax, or intracranial hemorrhage. This indicates that frequent repositioning based on real-time assessment is safe and well tolerated even in fragile neonates, provided that hemodynamic stability is ensured. These safety results align with earlier feasibility reports showing that LUS can be safely applied in preterm infants for daily lung evaluation (34). Thus, our results strengthen the case for routine LUS integration into NICU respiratory management protocols.

The strengths of this study include its randomized controlled design, rigorous diagnostic definition of VAP (15), and standardized operator training. The incorporation of multivariate logistic regression allowed adjustment for key confounders such as gestational age, SNAPPE-II score, and comorbidities (PPHN, TTN, ICH, HIE). The consistent direction of effect across primary and secondary outcomes further supports the robustness of the findings.

However, some limitations must be acknowledged. Because we excluded premature infants as the highest risk group for VAP, the generalizability of our findings is limited. This choice was made to minimize confusion and establish a concept validation in a more stable semester queue. Future trials should validate and adjust the suitability of this protocol for premature infants, particularly those with severe preterm birth or chronic lung disease. At the same time, the decision to change position was based on qualitative pattern recognition rather than a predefined quantitative lung ultrasound (LUS) score. While this approach allowed for dynamic, clinically intuitive adjustments, future studies should investigate the utility of standardized LUS scores to establish objective, reproducible thresholds for initiating positional therapy. The single-center design may limit generalizability, as infection control practices and nurse-to-patient ratios vary across institutions (31). The relatively small sample size limits power to detect rare but clinically relevant outcomes such as mortality. Operator dependency remains an intrinsic limitation of LUS; although interobserver variability was minimized through structured training and periodic quality control, complete elimination is difficult (10). Finally, we acknowledge the potential for surveillance bias. The LUS-guided group received more frequent pulmonary imaging, which may have facilitated earlier detection of lung pathology. To mitigate this, VAP diagnosis required meeting blinded, objective CDC criteria. However, we cannot fully separate the effect of enhanced monitoring from that of the positioning intervention itself, as they are integral components of the LUS-guided protocol (35).

Future multicenter, adequately powered trials are warranted to validate these findings and to refine the LUS-guided positioning protocol—particularly in extremely preterm infants and those with chronic lung disease (36). Combining LUS with emerging bedside technologies, such as electrical impedance tomography and automated ultrasound image analysis powered by artificial intelligence, could further enhance precision and reproducibility (37, 38). Furthermore, cost-effectiveness analyses and implementation studies are needed to integrate LUS-guided protocols into neonatal infection prevention bundles at the policy level (9, 39).

## Conclusion

This randomized controlled trial demonstrates that integrating lung ultrasound into postural management significantly reduces ventilator-associated pneumonia and shortens ventilation duration in mechanically ventilated neonates, without increasing adverse events. By enabling real-time, physiology-guided positioning to optimize lung aeration and secretion clearance, LUS provides a practical, radiation-free strategy for personalizing respiratory care. These findings support its consideration as a safe and effective adjunct to standard VAP prevention bundles in the NICU. Future multicenter studies should validate this approach in preterm populations and evaluate its long-term outcomes and cost-effectiveness to guide broader clinical adoption.

## Data Availability

The original contributions presented in the study are included in the article/Supplementary Material, further inquiries can be directed to the corresponding author/s.
