# Supplementary figures and images for: Lung ultrasound–guided positioning strategy for the prevention of ventilator-associated pneumonia in neonates
Source: Front Pediatr. 2026 Feb 2;14:1765924. doi: 10.3389/fped.2026.1765924 (PMC12907429; doi:10.3389/fped.2026.1765924)

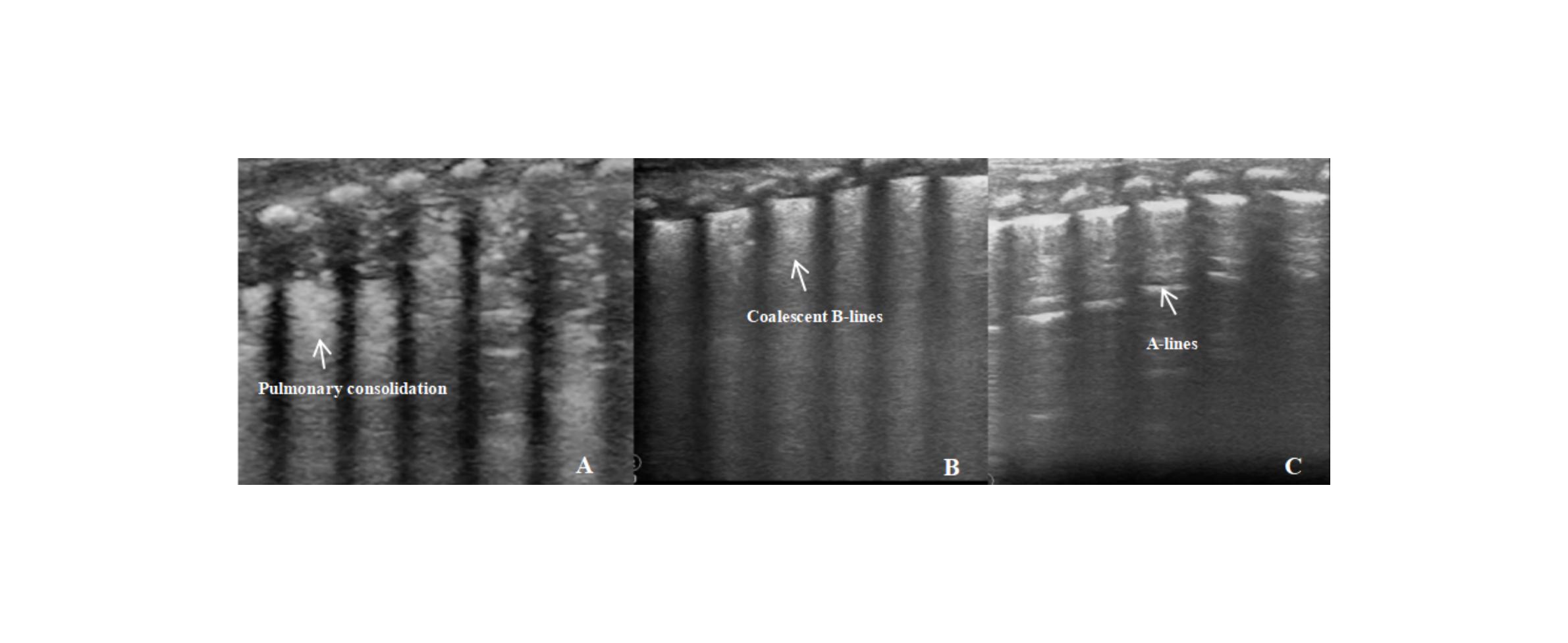

Supplement: Supplementary file 1 [file Image1.jpeg]

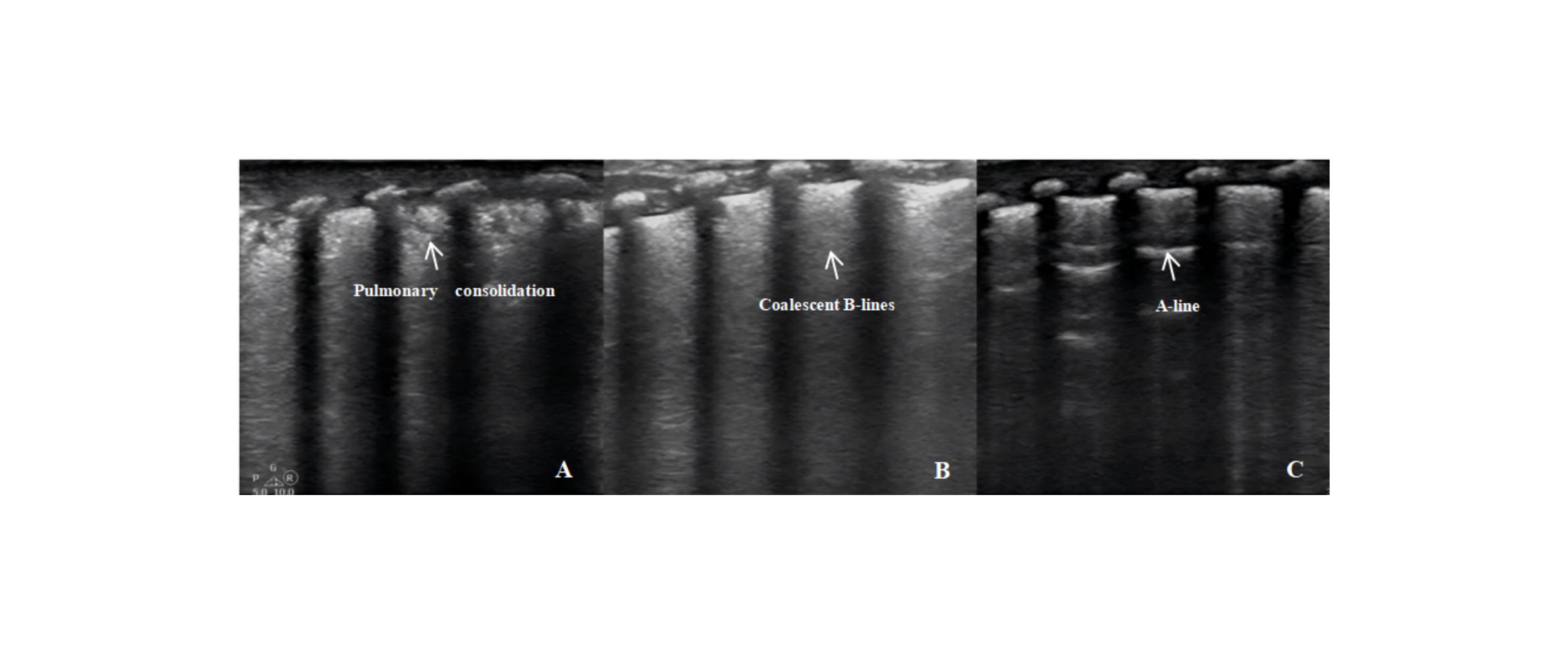

Supplement: Supplementary file 2 [file Image2.jpeg]
